# *Ephedra alte* (Joint Pine): An Invasive, Problematic Weedy Species in Forestry and Fruit Tree Orchards in Jordan

**DOI:** 10.1100/2012/971903

**Published:** 2012-05-03

**Authors:** Jamal R. Qasem

**Affiliations:** Department of Plant Protection, Faculty of Agriculture, University of Jordan, P.O. Box 13282, Amman 11942, Jordan

## Abstract

A field survey was carried out to record plant species climbed by *Ephedra alte* in certain parts of Jordan during 2008–2010. Forty species of shrubs, ornamental, fruit, and forest trees belonging to 24 plant families suffered from the climbing habit of *E. alte*. Growth of host plants was adversely affected by *E. alte* growth that extended over their vegetation. In addition to its possible competition for water and nutrients, the extensive growth it forms over host species prevents photosynthesis, smothers growth and makes plants die underneath the extensive cover. However, *E. alte* did not climb all plant species, indicating a host preference range. Damaged fruit trees included *Amygdalus communis, Citrus aurantifolia, Ficus carica, Olea europaea, Opuntia ficus-indica, and Punica granatum*. Forestry species that were adversely affected included *Acacia cyanophylla, Ceratonia siliqua, Crataegus azarolus, Cupressus sempervirens, Pinus halepensis, Pistacia atlantica, Pistacia palaestina, Quercus coccifera, Quercus infectoria, Retama raetam, Rhamnus palaestina, Rhus tripartita*, and *Zizyphus spina-christi*. Woody ornamentals attacked were *Ailanthus altissima, Hedera helix, Jasminum fruticans, Jasminum grandiflorum, Nerium oleander*, and *Pyracantha coccinea*. Results indicated that *E. alte* is a strong competitive for light and can completely smother plants supporting its growth. *A. communis, F. carica, R. palaestina*, and *C. azarolus* were most frequently attacked.

## 1. Introduction

The Ephedraceae family consists of species with varied growth forms, habits and habitat requirements. Shrubby species that belong to this family have erect stems, decumbent plants up to 1 m high; scarious leaf sheath, at least of young shoots, 1-2 mm long, as long as the diameter of the subtended stem and longer than leaf rudiments. Climbing or prostrate plants are often with long lignified stems but when forming a shrub (after grazing), the scarious sheath is usually shorter than the diameter of subtended stem or leaves. Fleshy ripe fruiting bracts are red; the free part of the leaf is mostly less than 3 mm long and flowering branchlets are always arise from thicker stems with green photosynthetic bark [1, 2].

*Ephedra *is a distinct genus that consists of 50–65 species among which are shrubs, vines, but rarely small trees [3–5]. It is a dioecious plant, heavily branched, with very short scale leaves. It is a nonsucculent glycophyte that grows in natural habitats and is widely distributed in temperate regions in different parts of the world but usually common in dry and open habitats and in the deserts. It has been reported at elevations ranging from the near sea level (species around the Mediterranean Sea) to almost 5000 m *(E. gerardiana *in the Himalayas). Under drought, heat, and frost conditions in highlands in Asia, species have shown greater wood xeromorphy than do the lowland species [6]. *Ephedra* is recorded in mobile and stable sand dunes and wadis with sandy ground and is heavily consumed by camels and other grazing animals.

*Ephedra* is long known for its medicinal value in the Mediterranean because of the ephedrine alkaloid and other chemicals in the stems of most members of this genus [7, 8]. Ephedrine has been long known to have contact allergenic properties and as being valuable in the treatment of asthma and many other complaints of the respiratory system [9]. Its naturally occurring isomer pseudoephedrine also appears as a contact sensitizer. However, recently *Ephedra*-derived products have been found hazardous and may cause cardiac dysfunction and even death when excessively used [9]. Other chemicals isolated from the aerial parts of *E. alte *were vicenin II, ephedralone, p-coumaric, protocatechuic acids, herbacetin [10], ephedradine C, and hordenine [11]. However, *Ephedra* spp. are widely varied in alkaloid content while these chemicals are generally absent in roots, berries, and seeds of these species.

*E. alte* (synonym *Ephedra aphylla* Forssk) is one of the common species in different Middle East countries [1]. It is normally a shrub not more than 1.4 m in height, but when it grows with taller vegetation, such as along irrigation ditches, it may grow into those plants as a scandent liana [2]. It is found hanging from cracks in limestone cliffs or near wadis in sand and is often found growing in juniper forest with *Pistacia, Opuntia, Daphne linearifolia, Artemesia,* and *Thymelaea hirsuta*. It flowers and fruits from March to June. This species extends across the eastern Mediterranean to the Arabian Peninsula [12] and is the only *Ephedra* species within its range from dry to very dry habitats that may be somewhat more extensive, from cliffs, along wadis, and with phreatophytes along irrigation ditches [2]. It is able to grow in dry habitats to which few angiosperms have adapted and has been mentioned as somewhat peculiar in its growth habit. *E. alte* has been also mentioned as being of a high toxicity against *Aedes aegypti* larvae and thus may become an important plant in controlling disease-causing mosquitoes [12].

In Jordan, four *Ephedra* species, *Ephedra alata* Decne, *Ephedra alte* C. A. Mey,* Ephedra foliate* Boiss., and *Ephedra transitoria* Riedl., have been reported [13] to spread in natural habitats and recorded in areas in and close to the Jordan valley (tropical and subtropical), the Mediterranean, and Sahara Arabian. *E. alte* has been mentioned to grow in arid stony desert where average annual rainfall is between 1 and 15 mm [14]. Contrary to other species, *E. alte* is mobile and found climbing many fruit and forestry species, which resulted in severe growth damage and death of these in different regions in the country. It may be regarded as an ecologically dangerous and a threatening species to the survival of many woody species. The literature on its negative ecological impact and behavior as an agricultural pest is lacking; therefore, the following was thought important:

- recording species occurrence,
- recording inflicted species by *E. alte* growth,
- quantifying the effect of *E. alte* on fruit species,
- visualizing any possible negative effects other than competition and smothering.

## 2. Materials and Methods

### 2.1. Study Procedure

This study aimed to survey *E. alte* in certain parts (covered by forestry and/or fruit trees) of Jordan at which this species was more frequently observed, record its growth status and woody plant species attacked, and accommodate its climbing habit. The survey was carried out during the period from 2008 to 2010 at which *E. alte* was recorded in cultivated fields, fruit tree orchards, forests, and range lands. The survey covered most regions in the central and northern parts of Jordan which located between 36°00′E longitude and 31°00′N latitude and included the central and northern Jordan Valley, Dead Sea, As-Salt, Wadi-Shu'aib, Zay, Baqqai, Nau'r, Amman, Ma'daba, Zarqa, Jerash, Irbid, and Ajloun, at which more than 150 vegetated sites were surveyed year round. The distance between sites was varied depending on the presence or absence of woody vegetation cover. At each site *E. alte* was checked and climbed host species were recorded. The bulk cover of vegetative mass of *E. alte* intermingled or laid over vegetation of other plants was subjectively rated as light, moderate, or high after being visually estimated in the field. Incidence was recorded as rare (found only on few plants in one geographical location or biogeographical region), limited (found on few plants localized in certain locations of 1 or 2 biogeographical regions), common (found on more than 10 plants in 1 or 2 biogeographical regions), or very common (found on many plants at multiple locations within more than two biogeographical regions). *E. alte* and plant species inflicted were all photographed. 

In late 2010, additional search was carried out at which six sites in different biogeographical regions were selected to represent the total surveyed locations (Table 1). In each site, *E. alte* was checked on the climbed host species. Species and number of plants climbed from each in every site were recorded. Frequency of the attacked number of plants of each species and between all sites was determined and percentage of their aerial parts covered by *E. alte* vegetation was visualized within and between sites. *E. alte* cover observed over climbed species was estimated using a cover abundance scale [15, 16]. Presence percentage was obtained from the number of *E. alte* plants in a specific site out of the six sites studied. Cover was estimated from estimates of *E. alte* vegetative mass projected on climbed tree as a percentage of total vegetative area of attacked species [17]. Climbed species frequency was used to detect changes in attacked number of plants in each species between different sites of different biogeographical regions. It is used to describe *E. alte* distribution on species forming plant community and often used in combination with density or cover estimates to measure trend or condition. 

 Notes on the vegetation type and dominating woody species in each site were also recorded. 

### 2.2. Statistical Analysis

Data on the number of plants attacked by *E. alte* from each species and the percentage coverage of their aerial parts by the climber vegetation within and between the selected sites were subjected to the analysis of variance (ANOVA) and performed on species, sites, and species by sites using GLM procedure of SAS [18]. Means of percentage coverage of each species within each site were separated for significance using the *t*-test at *P* ≤ 0.05 and frequency of the attacked number of plants and coverage percentage of species attacked were calculated using the Chi-square test using Freq procedure of SAS. 

## 3. Results

**Figure 1:**
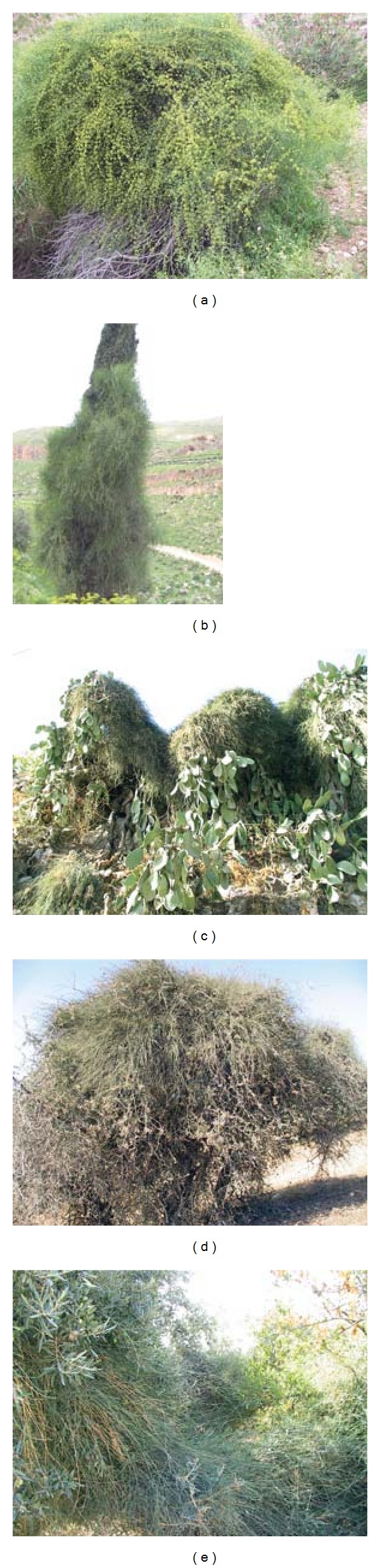
Joint-pine (a) killing *Amygdalus communis*, (b) climbing *Cupressus sempervirens,* (c) on *Opuntia ficus-indica*, (d) killing *Rhamnus palaestina,* and (e) climbing *Olea europaea*. Photos Scale: 23%  × 23%.

*E. alte* was found climbing/covering 40 plant species of fruit (9 species), and forest (13 species) trees, ornamentals (6 species) and shrubs (12 species) that belong to 24 plant families (Table 2). Wild and cultivated species smothered by *E. alte* included deciduous and evergreen species. Among severely affected fruit trees were *A. communis*,* C. aurantifolia*, *O. europaea*, *O. ficus-indica*, *Prunus persica,* and *P. granatum*. Peculiarly, *E. alte* was also found climbing other climbers including *H. helix* (a woody ornamental), *Jasminum* spp., *Galium* sp., *Asparagus stipularis,* and *V. vinifera*. Although *E. alte* attacked different species, but its growth development was substantially varied on different targets (Table 2). It formed a massive growth on *A. communis*, *O. ficus-indica,* and *R. palaestina* (Figure 1) but relatively smaller growth on *C. siliqua*, *P. halepensis*, *P. granatum,* and* Q. coccifera*. *E. alte * however, was destructive to* A. communis*,* C. azarolus*, *R. palaestina* and* Z. spina-christi*. It was more frequently observed on *A. communis*, *O. ficus-indica*, *R. palaestina,* and *C. azarolus* but rare on *H. helix*, *Jasminum *spp.,* P. coccinea, P. halepensis* and *V. vinifera *(Table 3). 

**Figure 2:**
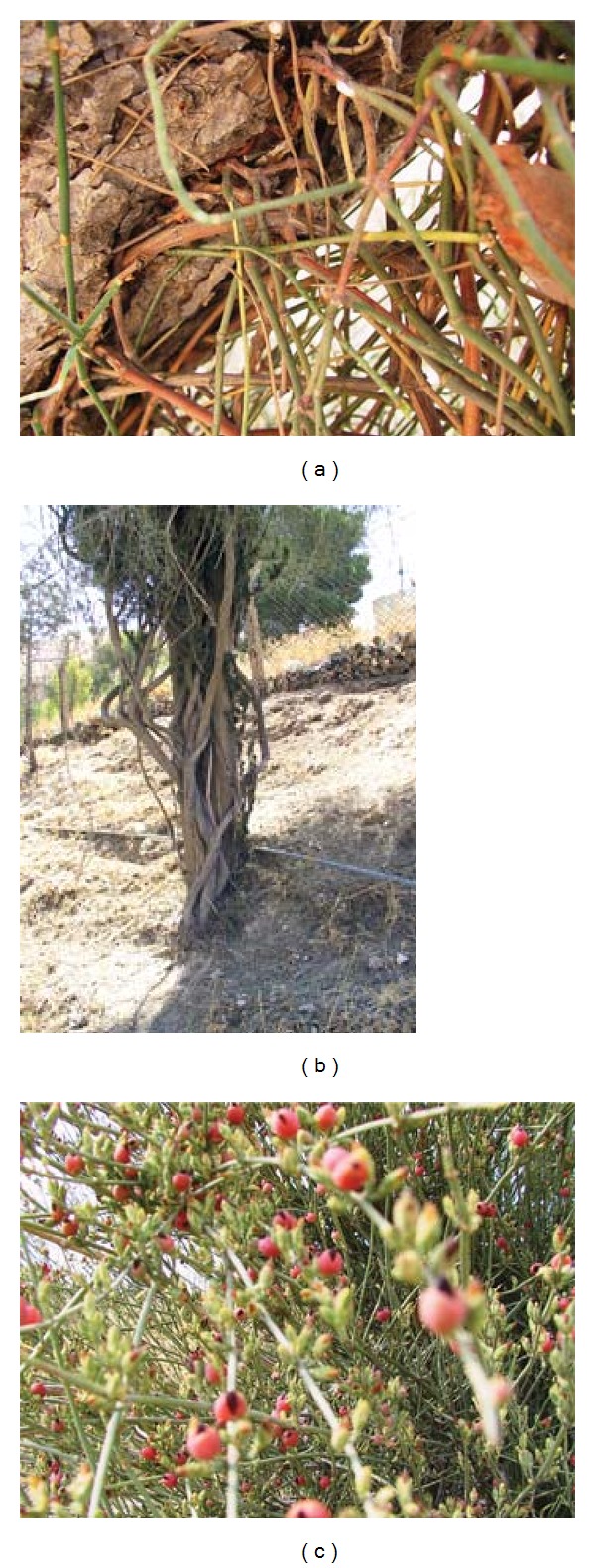
Joint-pine (a) aerial roots inserted in *Pinus halepensis* bark. (b) Emerged exactly with *Cupressus sempervirens var. horizontalis.* (c) Fruiting stage. Photos Scale: 36%  × 36%.

*E. alte* forms adventitious roots that enable it to climb and attach to stem and branches of host plant. These roots were found penetrating the cracked bark of old *P. halepensis* trees (Figure 2(a)). However, connection between these and internal host tissue was not observed indicating a commensalisms relationship. 

Considering representative sites surveyed late in 2010, certain species were heavily attacked by *E. alte* with the highest number of smothered plants. Among all species, *A. communis* was most frequently climbed with a total number of 87 plants in all sites (Table 3) followed by *R. palaestina* (67 plants) and *C. monogyna* (49 plants). However, species such as *A. stipularis, O. europaea, Q. coccifera. P. palaestina,* and *R. raetam* showed moderate numbers of climbed plants. Other species had less than 10 plants attacked. Frequency of *E. alte* occurrence was the highest on *A. communis, R. palaestina,* and *C. monogyna* in different sites (Table 3). However, *E. alte* vegetative cover was highly varied between climbed species with *P. persica, A. stipularis, Galium *sp*., I. viscose,* and *C. monogyna* showing the highest coverage frequency by *E. alte* vegetation. 

*A. communis* and *R. palaestina* were attacked in all sites (Table 4) followed by *O. europaea* and *C. spinosa* (in five sites). Differences in species number and vegetative cover frequency by *E. alte* were found between and within the searched sites (Table 4). 

## 4. Discussion

Four *Ephedra* species have been reported to occur in Jordan [13, 19], and found growing in a wide range of habitats.* E. alte* appeared the most problematic since rapidly spread, invading and climbing both forest and fruit tree species in different locations. It grows at elevations from 255 m b.s.l to 1500 m a.s.l. and found in humid to dry and very dry regions to which few plant species have adapted. It was reported to extend across the eastern Mediterranean to the Arabian Peninsula. 

Although *E. alte* grows separately in open lands sparsely or hardly covered by vegetation, it tends strongly to climb other plant species in its surroundings. It emerges beside other woody species (Figure 2(b)), climbs them, and rapidly forms a massive vegetative growth with long rope-like stems extending over aerial parts of other species. *E. alte* has been reported as being of unusual morphology among the North American and the European-Mediterranean species in having a strongly climbing liana habit [4] and a relatively unusual morphological feature of partially twining habit [6, 20]. Thus it resembles many weed species in that its stems twining on the stem of climbed plant and its branches are extended from on, or in between those of host plants, compete for light, prevent photosynthesis, and become difficult to control by none highly selective herbicides or even through hand removal. Therefore, *E. alte* may be regarded as an aggressive weed that must be controlled if to avoid its damage to other species. *Ephedra* has been reported as a unique genus among gymnosperms in its high frequency of polyploidy found in about 65% of species studied [21], including 22% of species in which both diploid and polyploidy counts have been obtained. This, however, is a character shared with many noxious weed species. *E. alte,* however, is considered as a weed in Egypt [12]. 

It is well established that weeds compete for water, light, and nutrients [22]. In dense forests, light may become very limited in quantity and quality and far-reaching short stature species or low vegetation layers. It seems that *E. alte* with a unique vegetative growth (scale-like leaves, lignified stems, massive growth, etc.) tends to climb other species, forming a mattress-like vegetative cover, making it difficult for the attacked plants to perform normal photosynthesis and producing enough food to maintain growth and survive under poor fertility and low light supply. *E. alte;* however, it does not only prevent light from reaching effected species but its multiple stems emerge from the same point of host stem emergence (Figure 2(b)). This growth habit seems unusual, not fully understood [6], and somehow similar to that of certain parasitic species (e.g., *Cuscuta* spp.), while its tendency to grow alone as well as to climb other species is the same as that of certain hemiparasitic genera including *Osyris *and *Thesium* [23]. These parasites also grow separately, do photosynthesis, but tend strongly to parasitize other plant species. In addition, the shape, appearance, and structure of *E. alte* fruits more or less resemble those of some parasitic genera (*Viscum*, *Loranthus,* and *Osyris*). Fruit is a berry, enclosing a single seed surrounded by a sticky juicy bulb that facilitates dispersal by birds (Figure 2(c)). 

Emergence of *E. alte* stem from the near host stem may question its self-dependence for food, nutrients, and/or water. *E. alte* has scale-like leaves, its photosynthate area is mainly the green stems extended over vegetative parts of host species, but photosynthetic materials produced may not be high enough to support the bulky growth it forms in dense-thick plant populations. Shoots of some plants of* E. alte* were found reddish in color which may indicate deficient mineral element/s or low chlorophyll content and thus photosynthate materials produced. However, its climbing habit frequently occurred under both dense and sparse plant populations. 

Although no connection was detected between aerial parts of *E. alte* and attacked species, adventitious roots developed from stem nodes of this species were found inserted in the cracked bark of *P. halepensis* trees and sometimes hard to pull out from host tissues (Figure 2(a)). In addition, extension of *E. alte* stems beside host stem may suggest certain sort of connection or interrelationship between their root systems. This hypothesis, however, was not examined in the present work since *E. alte* roots were deeply extended in rocky soils. This may remain possible in form of natural root crafting which could be tested by injecting a suitable translocated herbicide into the stem of climbed species and following up any changes that may occur on growth of *E. alte*. It may be also examined by growing *E. alte* with a preferred and usually climbed species in a container for certain period and examining their root systems. The ambiguity in the rooting among the major groups of *Ephedra *is also evidenced [24] and is only likely to be solved by an examination of a number of divergent sequence regions to obtain an adequate number of informative characters. There is a limited degree of correlation between putative derived character states such as dry, winged ovulate cone bracts, single seed per cone, or unusual habit types, suggesting considerable homoplasy in the genus [6]. 

*E. alte* seems to have host preference. It climbed *C. aurantifolia *but was rarely found on* Citrus limon *and not recorded on *Citrus orange* while the surrounding* C. sempervirens* plants were attacked. In contrast, *E. alte* was not observed on* Casuarina equisetifolia* (Australian pine), *Tamarix pentandra* (tamarisk), or* Melia azedarach* (Chinaberry) trees. It attacked *Z. spina-christi* but not *Zizyphus jujuba* (common jujube). The relatively high number and diversity of targeted species may suggest certain type of association, high phenotypic plasticity, and/or physiological adaptability. Compatibility with chemicals that some of these targeted species may release into the surrounding environment is another possibility although was not tested in the present work. Released chemicals may attract or repel *Ephedra* from climbing certain species and thus subsequently enhance or inhibit its growth. Root exudates may also have a role in whether consist beneficial/harmful compounds that stimulate/inhibit emergence and growth of *E. alte* seedlings nearby and close to these species. Root secretions may contain certain growth promoters or mineral nutrients that stimulate growth of *E. alte* and explain its association with certain woody species but not others. Although *Ephedra* spp. were not reported as parasites but their morphology, growth habit, and maybe responses to certain conditions are more or less similar to those of certain parasitic species. Some parasitic species are also stimulated to germinate and to grow by chemicals released from roots of their host plants. *E. alte* was found completely destructive to many attacked species, and its ultimate effect on host plants is more or less similar to that of different parasitic genera (*Cuscuta*, *Loranthus,* and* Viscum*). Moreover, similarity between *E. alte* and these species may be also demonstrated through the long-distance seed dispersal that is probably mediated by migratory birds [25] although the potential for overwater dispersal is also evidenced by its wide spread distribution [3]. 

Variations in growth of *E. alte* on different plant species may reflect differences in ecological adaptation, chemical, morphological, or physiological compatibility between these and *E. alte*. 

Studies on *Ephedra* spp. control are lacking since they are not considered as weeds. However, *Ephedra* control may be achieved by cutting the bulky stems just emerging from the above soil level; this possibility, however, was not employed in the present work. *E. alte* was also found attacked by different natural enemies in Jordan including mille bugs and a scale insect from croccideae (Hemopterae) (unpublished data) which led to desiccation of its green stems. Injection of *E. alte* by a suitable translocated herbicide may be another control option while foliage application of herbicides can be practiced on separate *Ephedra* plants but not after climbing other species; otherwise low selective rates may be used or a directed treatment to avoid host injury. This, however, could be a future research line with other aspects of *E. alte* associations, host preference within and among plant species, male and female aggressiveness, and climbing habit in relation to indigenous chemicals of inflicted species. Some work on the competitive relationships between *E. alte* and its climbed species over nutrients and water is worth conducting. 

It is worth indicating that this work is first of its kind in the country and at world level that treated *E. alte* as an agricultural pest of a serious threat to other woody species of different growth habit or forms. The potential harmful effect of this species on others in its vicinity was even not thought about by researchers before the conduction of this work. The present study is the first to predict possible negative association/interrelationship (through root crafting or other types) of this species with a large number of economic or ecologically important woody species in certain form of dependence on other species. In addition, *E. alte* is for the first time considered as a strong smothering weedy species that competes for light and maybe nutrients and/or water with host species and proved highly successful in dense as well as in sparse vegetation under different environments. 

In conclusion, *E. alte* may be regarded as of a potential threat to different fruit and forestry species in Jordan. Its ecological harmful effects may be serious considering that forestry area represents less than 1% of the total country's area while almost 90% is a desert not receiving more than 50–70 mm average annual rainfall. The ecological threat this species exerts on other plant species becomes clearer when interacting with other ecological stresses, for example, poor soil fertility, frequent grazing, fire hazard, drought, and housing activities. All exert an ecological stress on the existence of certain forestry species or on the area devoted to fruit trees plantation. However, there is still much information required, and more studies are needed on *E. alte* prevalence in other parts of the country. Questions on climbing habit of this species in relation to ecological, physiological, and biochemical interactions with target species remained to be addressed, while its ecological threat and severity of this effect under different ecological conditions merit further research. 

## Figures and Tables

**Table 1:** Representative sites description and plant number attacked by *E. alte* late in 2010 survey.

No.	Site name	Biogeographical region	Total plants climbed by *E. alte* per site	Estimated area checked (ha)	Approximate latitude (m above sea level)	Common vegetation
(1)	Amman	Mediterranean	23	4	1000	*Olea europaea*
(2)	Amman	Mediterranean	168	6	1000	*Olea europaea*
(3)	Zarqa	Mediterranean	26	5	900	*Olea europaea*
(4)	Jerash	Mediterranean	49	4	1100	*Citrus* spp.
(5)	Wadi-Shu'aib upper and As-Salt	Subtropical-Mediterranean	46	3	−300–750	*Olea europaea*, *Citrus* spp. and almonds
(6)	South Shuna and central Jordan Valley	Tropical	49	5	−255	*Citrus* spp. and *Zizyphus spina-christi* and mixtures

**Table 2:** Common, scientific, and family names, growth form, vegetative mass, incidence, and biogeographical regions of plant species attacked by *E. alte* in Jordan for the period 2008–2010.

Common name	Scientific name	Family name	Growth status	Vegetative mass of *E. alte *	Incidence	Biogeographical region
Fruit trees						

Almond	*Amygdalus communis *L.	Rosaceae	C and W	High	Common	Subtropical and Mediterranean
Mexican lime	*Citrus aurantifolia* (Christm.) Swingle	Rutaceae	C	High	Limited	Mediterranean
Lemon	*Citrus limon* L.	Rutaceae	C	Moderate	Rare	Mediterranean and Subtropical
Fig	*Ficus carica* L.	Moraceae	C	High	Limited	Mediterranean and Subtropical
Grape	*Vitis vinifera* L.	Vitaceae	C	Moderate	Rare	Mediterranean
Indian fig	*Opuntia ficus-indica* (L.) Miller	Cactaceae	C	High	Very common	Mediterranean, tropical, and Subtropical
Nectarine	*Prunus persica *L.	Rosaceae	C	High	Rare	Mediterranean
Olive	*Olea europaea *L.	Oleaceae	C	High	Limited	Mediterranean
Pomegranate	*Punica granatum* L.	Punicaceae	C	High	Limited	Subtropical

Shrubs						

Bedstraw	*Galium *sp.	Rubiaceae	W	High	Rare	Mediterranean
Fern-leaved clematis	*Clematis cirrhosa *L.	Ranunculaceae	W	Moderate	Rare	Mediterranean
Giant cane	*Arundo donax* L.	Gramineae	W	Light	Rare	Subtropical
Grey asparagus	*Asparagus stipularis* Forssk.	Liliaceae	W	Moderate	Rare	Mediterranean
Caper	*Capparis spinosa* L.	Capparidaceae	W	High	Rare	Subtropical
Indian fleabane	*Pluchea indica* (L.) Less.	Compositae	W	Moderate	Rare	Tropical and Subtropical
Inula	*Inula viscosa *(L.) Aiton	Compositae	W	Light	Rare	Subtropical
Jerusalem spurge	*Euphorbia hierosolymitana* Boiss	Euphorbiaceae	W	Light	Rare	Mediterranean
Syrian mesquite	*Prosopis farcta* (Banks and Soland.) J. F. Macbr	Leguminosae	W	Moderate	Rare	Subtropical
Sumac	*Rhus coriaria* L.	Anacardiaceae	W	High	Rare	Subtropical
Sumac	*Rhus tripartita* L.	Anacardiaceae	W	Moderate	Limited	Subtropical
Thorny burnet	*Sarcopoterium spinosum *(L.) Spach	Rosaceae	W	High	Rare	Mediterranean

Ornamental shrubs and climbers						

Common ivy	*Hedera helix* L.	Araliaceae	C	High	Rare	Mediterranean
Heaven tree	*Ailanthus altissima* (Mill.) Swingle	Simaroubaceae	C	Moderate	Rare	Mediterranean
Bush jasmine	*Jasminum fruticans* L.	Oleaceae	C	Light	Rare	Mediterranean
Jasmine	*Jasminum grandiflorum* L.		C	High	Rare	Mediterranean
Oleander	*Nerium oleander* L.	Apocynaceae	W	Light	Rare	Subtropical
Firethorn	*Pyracantha coccinea *Roem	Rosaceae	C	Light	Rare	Mediterranean

Forest trees						

Aleppo oak	*Quercus infectoria* Olivier	Fagaceae	W	High	Limited	Mediterranean
Aleppo pine	*Pinus halepensis *Mill.	Pinaceae	C	Light	Rare	Mediterranean
Carob	*Ceratonia siliqua* L.	Leguminosae	C	Moderate	Rare	Subtropical
Christ thorn jujube	*Zizyphus spina-christi* L.	Rhamnaceae	W	High	Very common	Subtropical
Cypress	*Cupressus sempervirens *L.* var. horizontalis *(Miller) Gordon	Cupressaceae	C	High	Limited	Mediterranean
Cypress	*Cupressus sempervirens *L.* var. pyramidalis *	Cupressaceae	C	High	Limited	Mediterranean
Golden wreath wattle	*Acacia cyanophylla* Lindley	Leguminosae	C	Moderate	Limited	Subtropical
Hawthorn	*Crataegus monogyna* Jacq.	Rosaceae	W	High	Very common	Mediterranean
Kermes oak	*Quercus coccifera *L.	Fagaceae	W	High	Very common	Mediterranean
Mosphilla	*Crataegus azarolus* L.	Rosaceae	W	High	Very common	Mediterranean
Palestine buckthorn	*Rhamnus palaestina* Boiss	Rhamnaceae	W	High	Very common	Subtropical
Terebinth	*Pistacia atlantica* Desf.	Anacardiaceae	W	High	Rare	Mediterranean
White weeping broom	*Retama raetam *(Forskal) Webb and Berth	Leguminosae	C	High	Common	Subtropical and Mediterranean
Wild pistachio	*Pistacia palaestina *Boiss	Anacardiaceae	C	Moderate	Common	Mediterranean

Rare: only on few plants in 1-2 sites of a biogeographical region.

Limited: on few plants localized in certain locations of 1 or 2 biogeographical regions.

Common: on certain plant species in > one biogeographical region.

Very common: on many plant species in different locations of different biogeographical regions.

C: cultivated, W: wild.

**Table 3:** Total number and frequency of *E. alte* plants per host plant species attacked and coverage percentage on host plant in six randomly selected representative sites in Jordan late in 2010.

Plant species	Frequency of *E. alte* density (plants per host species)	Frequency of *E. alte* density (%)	*E. alte* coverage (%)
Simaroubacea *A. altissima *	2	0.55	77.0 ± 15.5 cd*
Rosaceae *A. communis *	87	24.10	77.9 ± 3.0 cd
Gramineae *A. donax *	1	0.28	67.0 ± 21.5 bcd
Liliaceae *A. stipularis *	10	2.77	88.3 ± 7.0 d
Capparidaceae *C. spinosa *	8	2.22	45.5 ± 7.7 ab
Rutaceae *C. limon *	1	0.28	24.9 ± 21.9 ab
Cupressaceae *C. sempervirens *L.* var. pyramidalis *	7	1.94	55.6 ± 8.9 abc
Rosaceae *C. monogyna *	49	13.57	81.1 ± 3.7 cd
Rutaceae *C. aurantifolia *	5	1.39	35.0 ± 10.3 ab
Euphorbiaceae *E. hierosolymitana *	8	2.22	71.2 ± 7.9 bcd
Moraceae *F. carica *	5	1.39	69.9 ± 9.8 bcd
*Rubiaceae * *Galium* sp.	1	0.28	87.0 ± 21.5 cd
Compositae *I. viscosa *	1	0.28	84.1 ± 21.5 cd
Apocynaceae *N. oleander *	1	0.28	14.9 ± 21.9 a
Oleaceae *O. europaea *	19	5.26	46.8 ± 5.2 ab
Cactaceae *O. ficus-indica *	4	1.11	72.9 ± 11.3 cd
Pinaceae *P. halepensis. *	6	1.66	52.0 ± 8.9 abc
Compositae *P. indica *	2	0.55	49.9 ± 16.0 abc
Rosaceae *S. spinosum *	5	1.39	79.2 ± 9.8 cd
Leguminosae *P. farcta *	4	1.11	72.7 ± 11.0 cd
Rosaceae *P. persica *	1	0.28	98.0 ± 21.5 d
Anacardiaceae *P. palaestina *	10	2.77	56.5 ± 7.0 abc
Punicaceae *P. granatum *	3	0.83	68.7 ± 12.5 bcd
Fagaceae *Q. coccifera *	15	4.16	79.4 ± 6.4 cd
Leguminosae *R. raetam *	26	7.20	58.9 ± 6.7 abc
Rhamnaceae *R. palaestina *	67	18.56	78.2 ± 3.1 cd
Anacardiaceae *R. coriaria *	2	0.55	54.1 ± 15.5 abc
Anacardiaceae *R. tripartita*.	4	1.11	47.4 ± 12.0 ab
Rhamnaceae *Z. spina-christi *	7	1.94	73.5 ± 9.8 cd

*Means within column followed by the same letter were not significantly different according to *t*-test at *P* ≤ 0.05.

Numbers of % coverage represent mean values **±** SE.

**Table 4:** Distribution of climbed plant species by *E. alte* between six representative sites selected randomly in Jordan, showing percentage of climber number and their coverage percentage on each species and between sites late in 2010.

Plant species	Site 1		Site 2		Site 3		Site 4		Site 5		Site 6	
	No. of *E. alte* (%)	Cover by *E. alte* (%)	No. of *E. alte* (%)	Cover by *E. alte* (%)	No. of *E. alte* (%)	Cover by *E. alte* (%)	No. of *E. alte* (%)	Cover by *E. alte* (%)	No. of *E. alte* (%)	Cover by *E. alte* (%)	No. of *E. alte* (%)	Cover by *E. alte* (%)
* A. altissima*	—	—	—	—	—	—	4.1	80.0 abc	—	—	—	—
* A. communis *	13.0	80.0 b*	35.7	76.6 b	42.3	83.3 b	4.1	64.0 abc	21.7	73.0 ab	2.0	95.0 c
* A. donax*	—	—	—	—	—	—	2.0	70.0 abc	—	—	—	—
*A. stipularis*	8.7	85.0 b	4.2	90.4 b	—	—	—	—	2.2	50.0 ab	—	—
*C. spinosa*	—	—	0.6	5.0 a	3.9	10.0 a	4.1	50.0 ab	2.2	95.0 b	6.1	66.7 abc
* C. lemon *	—	—	—	—	—	—	—	—	—	—	2.0	40.0 b
*C. sempervirens*	—	—	—	—	—	—	14.3	58.6 abc	—	—	—	—
* C. monogyna*	34.8	67.0 b	20.8	82.2 b	19.2	71.6 b	2.0	100.0 c	—	—	—	—
* C. aurantifolia*	—	—	—	—	—	—	10.2	38.0 a	—	—	—	—
* E. hierosolymitana*	—	—	4.8	71.3 b	—	—	—	—	—	—	—	—
* F. carica*	—	—	—	—	—	—	6.1	56.7 abc	4.4	90.0 b	—	—
* Galium* sp.	—	—	—	—	—	—	2.0	90.0 c	—	—	—	—
* I. viscosa *	—	—	—	—	—	—	—	—	2.2	80.0 ab	—	—
* N. oleander*	—	—	—	—	—	—	—	—	—	—	2.0	30.0 a
* O. europaea *	4.4	15.0 a	3.0	47.0 a	3.9	20.0 a	20.4	59.5 abc	—	—	4.1	35.0 a
* O. ficus-indica*	—	—	—	—	—	—	—	—	8.7	68.8 ab	—	—
* P. halepensis *	—	—	3.0	47.0 a	—	—	2.0	80.0 abc	—	—	—	—
* P. indica*	—	—	—	—	—	—	—	—	—	—	4.1	65.0 abc
* S. spinosum *	8.7	65.0 b	1.8	80.0 b	—	—	—	—	—	—	—	—
* P. farcta*	—	—	—	—	—	—	6.1	73.3 abc	—	—	2.0	95.0 c
* P. persica*	—	—	—	—	—	—	—	—	2.2	98.0 b	—	—
* P. palaestina *	8.7	12.0 a	—	—	—	—	8.2	72.5 abc	8.7	55.0 ab	—	—
* P. granatum*	4.4	95.0 b	—	—	—	—	—	—	4.4	45.0 a	—	—
* Q. coccifera *	—	—	—	—	—	—	—	—	28.3	76.8 ab	4.1	85.0 bc
* R. raetam *	—	—	—	—	—	—	4.1	40.0 a	—	—	49.0	75.8 bc
* R. palaestina*	17.4	62.5 b	26.2	80.3 b	30.8	81.0 b	10.2	69.2 abc	10.9	64.0 ab	2.0	95.0 c
* R. coriaria*	—	—	—	—	—	—	—	—	4.4	50.0 ab	—	—
* R. tripartita*	—	—	—	—	—	—	—	—	—	—	8.2	62.5 b
* Z. spina-christi*	—	—	—	—	—	—	—	—	—	—	14.3	88.6 c

*Means within column followed by the same letter were not significantly different according to *t*-test at *P* ≤ 0.05.

Numbers of % coverage represent mean values **±** SE.
